# Epidemiology of cervical cancer in Colombia

**Published:** 2012-12-30

**Authors:** Nubia Muñoz, Luis Eduardo Bravo

**Affiliations:** a National Cancer Institute, Bogota, Colombia, E-mail: Nubia.Munoz@free.fr; b Director Cancer Registry of Cali, Universidad del Valle, Cali, Colombia. E-mail: bravo.luiseduardo@gmail.com

**Keywords:** Cervix uteri cancer, HPV, HPV vaccines, cancer epidemiology, Cali, Colombia

## Abstract

Worldwide, cervical cancer is the third most common cancer in women, and the first or second most common in developing countries. Cervical cancer remains in Colombia the first cause of cancer mortality and the second cause of cancer incidence among women, despite the existence of screening programs during the last 3 decades. Bucaramanga, Manizales and Cali reported rates around 20 per 100,000and Pasto 27 per 100,000. The Cali cancer registry has reported a progressive decrease in the age standardized incidence and mortality rates of cervical cancer over the past 40 years. Reasons for the decline in incidence and mortality of cervical cancer are multiple and probably include: improvement in socio-economic conditions, decrease in parity rates and some effect of screening programs.

Human papilloma Virus is the main cause of cervical cancer, HPV natural history studies have now revealed that HPVs are the commonest of the sexually transmitted infections in most populations. Most HPV exposures result in spontaneous clearance without clinical manifestations and only a small fraction of the infected persons, known as chronic or persistent carriers, will retain the virus and progress to precancerous and cancer. HPV 16 and 18 account for 70% of cervical cancer and the 8 most common types. (HPV 16, 18, 45, 33, 31, 52, 58 and 35) account for about 90% of cervical cancer. Case-control studies also allowed the identification of the following cofactors that acting together with HPV increase the risk of progression from HPV persistent infection to cervical cancer: tobacco, high parity, long term use of oral contraceptives and past infections with herpes simplex type 2 and *Chlamydia trachomatis*. The demonstration that infection with certain types of human papillomavirus (HPV) is not only the main cause but also a necessary cause of cervical cancer has led to great advances in the prevention of this disease on two fronts: (i) Primary prevention by the use of prophylactic HPV vaccines; and (ii) secondary prevention by increasing the accuracy of cervical cancer screening.

## Abstract (es)

El cáncer de cérvix uterino es la tercera causa más frecuente de cáncer en el mundo y la primera o segunda más común en países en desarrollo. El cáncer de cérvix persiste en Colombia como la primera causa de mortalidad por cáncer en las mujeres, a pesar de la existencia de programa de tamización durante las últimas tres décadas. Bucaramanga, Manizales y Cali reportan tasas de incidencia alrededor de 20 por 100,000 y Pasto, 27 por 100,000. El Registro poblacional de cáncer de Cali ha reportado un descenso progresivo en las tasas de incidencia y mortalidad por cáncer de cérvix durante los últimos 40 años. Las razones para la declinación de las tasas de incidencia y mortalidad por cáncer de cérvix son múltiples y probablemente incluyen: mejoría en las condiciones socio-económicas, descenso en las tasas de fecundidad y algún efecto de los programas de tamización.

El virus del papiloma humano (VPH) es la principal causa de cáncer de cuello uterino. Los estudios de la historia natural del VPH han revelado que el VPH es la infección de transmisión sexual más común en la mayoría de las poblaciones. La exposición a VPH resulta en una resolución espontánea sin manifestaciones clínicas y únicamente una pequeña fracción de las personas infectadas, conocida como portadores crónicos, retendrá el virus y progresará a lesiones precursoras y cáncer.

Los genotipos de VPH 16 y 18 son responsables del 70% del cáncer de cérvix y los 8 tipos más comunes (VPH 16, 18, 45, 33, 31, 52, 58 and 35), se asocian con cerca del 90% de todos los casos de cáncer cervical. Los estudios de casos y controles también han permitido identificar los siguientes cofactores que actúan junto con la infección con VPH para aumentar el riesgo de cáncer de cérvix: tabaco, multiparidad, uso prolongado de contraceptivos orales y antecedentes de infecciones por virus del herpex simple tipo 2 y *Clamidya trachomatis. *

La demostración de que la infección por ciertos tipos de virus del papiloma humano (VPH) no sólo es la causa principal, sino, también, una causa necesaria del cáncer cervical, ha conducido a grandes avances en la prevención de esta enfermedad en dos frentes: (i) La prevención primaria mediante el uso de vacunas profilácticas contra el VPH, y (ii) La prevención secundaria mediante el aumento de la precisión de la detección del cáncer de cuello uterino.

## Introduction

Worldwide, cervical cancer is the third most common cancer in women, and the first or second most common in developing countries. From a total of 530,232 new cases that were estimated to have occurred in the world in 2008, 453,531 cases (86%) were diagnosed in less developed countries^1^. Its main public health importance in these countries lies in the fact that it affects relatively young and poor women, devastating not only the women themselves, but also their families. In Latin America cervical cancer is the second most common cancer among women (after breast cancer) and it is the most important cause of years of life lost, despite the fact that it is a highly preventable disease. It is estimated that if the prevention programs are not improved in the region, the annual number of cases diagnosed will increase from 68,000 cases in 2008 to 126,000 in 2025^2^.

Cervical cancer remains in Colombia the first cause of cancer mortality and the second cause of cancer incidence among women, ^3^ despite the existence of screening programs during the last 3 decades. The reasons for this lack of impact have been recently analyzed and include: poor quality of cytology, low coverage, especially of women at high risk and lack or partial follow-up of women with abnormal cytology ^4^

The highest mortality rates are observed in the most deprived regions ( along the main rivers, harbors, cities in the country borders) ^5^. In 2008 a total of 4,736 new cases and 2,154 deaths were estimated to have occurred. These numbers correspond to an age-adjusted incidence rate of 21.5 per 100,000 and a mortality rate of 10.0 per 100,000).^1^

**Figure 1.**
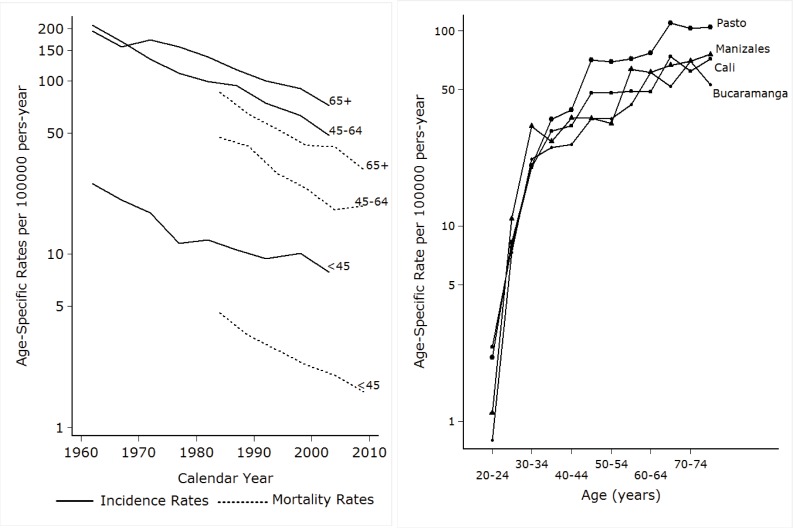
A.B A. Time trends of incidence and mortality rates of cervical cancer in Cali- Colombia, 1962 -2010 Fig. B Age-specific incidence rates of cervical cancer in four population-based Cancer Registries of Colombia. 2003 - 2007

There are now five population-based cancer registries in Colombia (Cali, Bucaramanga, Barranquilla, Manizales, and Pasto) and the one in Cali is the oldest in Latin America. Table 1 summarizes the age-adjusted incidence rates in 4 of these population-based cancer registries of Colombia. Bucaramanga, Manizales and Cali reported rates around 20 per 100,000and Pasto 27 per 100,000. ^6^ ^,^ ^7^ From 35 years onwards, the age specific incidence rates in Pasto are higher than in the other registries (Fig 1A).

**Table 1:**
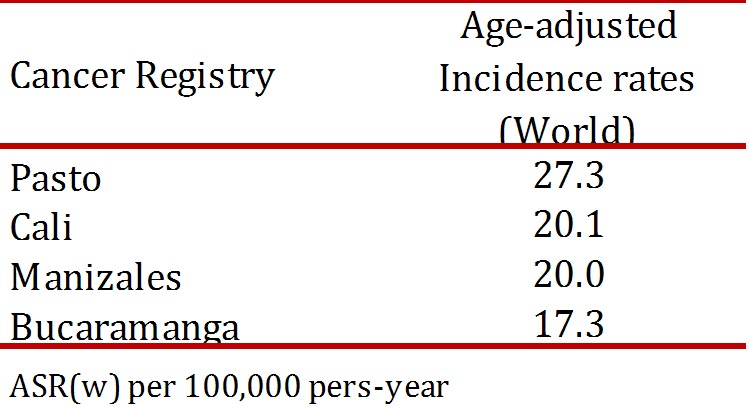
Cervical cancer incidence in population-based cancer registries of Colombia, 2003-2007

## Incidence and mortality in Cali

8.963 new cases of cervical cancer were registered in the population-based Cali cancer registry from 1962 to 2007. A total of 91.2% of these cases were diagnosed histologically and for only 3.9% the diagnosis was based on death certificates. During many years it was the most common cancer in Cali women, but it is now (2003-2007) the second most common cancer after breast cancer, with an age standardized rate (ASR) of 20.1 per 100,000 women. The mean age at diagnosis increased from 48.9 years (95% CI: 47.6 - 50.3) during 1962 to 1967 to 53.1 years (95% CI: 52.1- 54.2) in 2003-2007.

During the period of 1984 to 2011 a total of 2,595 women died from cervical cancer and the age standardized mortality rate in 2009 to 2011 was 7.0 per 100,000 women.

## Time trends

The Cali cancer registry has reported a progressive decrease in the age standardized incidence rates of cervical cancer from rates over 70 per 100,000 in 1960s to 20.1 in the period of 2003 to 2007. The annual decrease between 1962 and 2007 was 2.9%. This decrease was observed in all age groups and it was higher in the age group 45 - 64 years (3.2% annual decrease).The mortality rates decreased from 18.5 per 100,000 in 1984-88 to 7.0 during 2009- 2011, with an annual decrease of 4.2%. (Table 2 and Fig 1B)

Reasons for the decline in incidence and mortality of cervical cancer are multiple and probably include: improvement in socio-economic conditions, decrease in parity rates and some effect of screening programs.

**Table 2:**
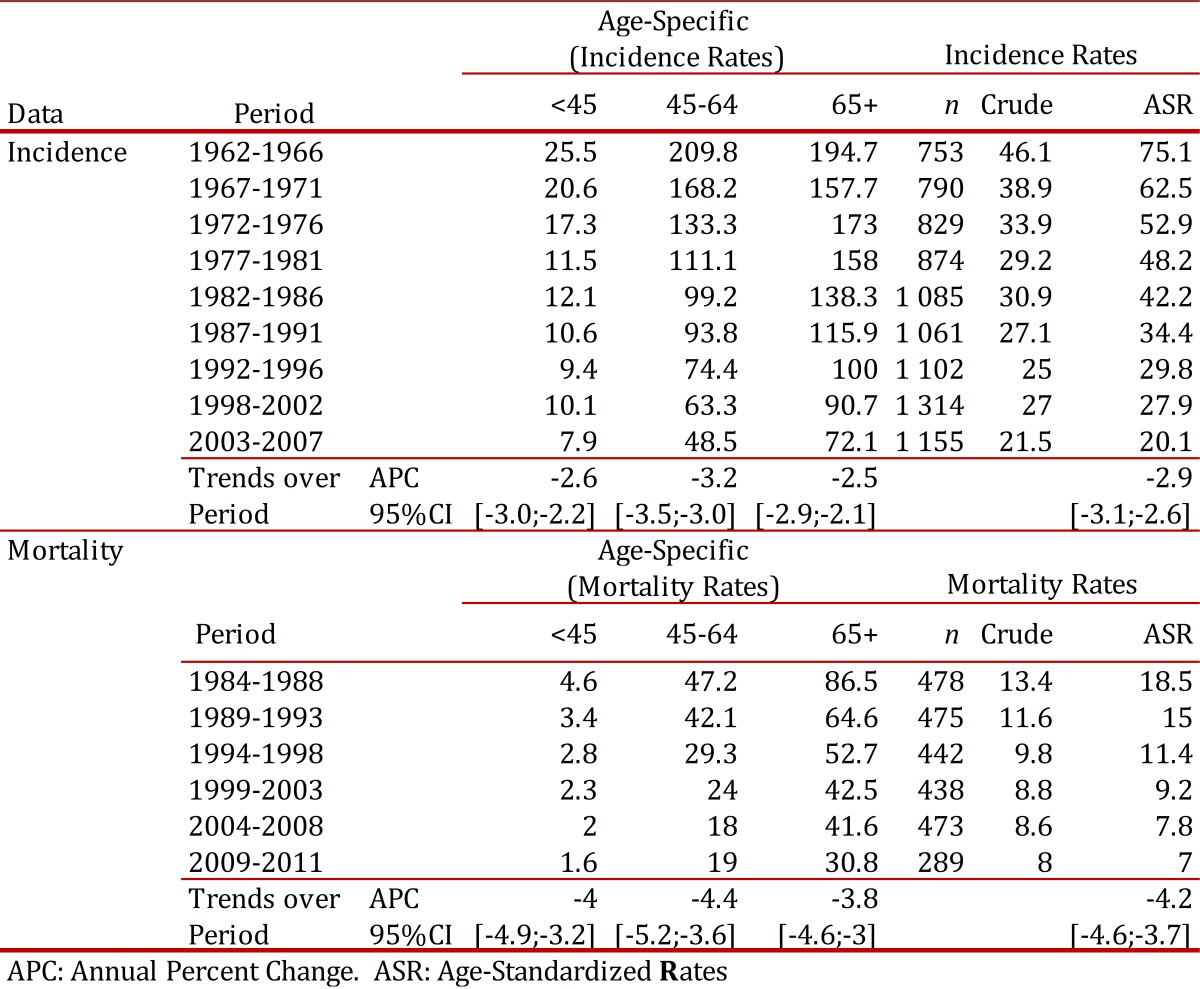
Cali, Colombia. Trends in Age-Specific Incidence Rates and Mortality Rates for Cervix Uteri Cancer Invasive Among Females, from 1962 to 2011.

**Figura 2:**
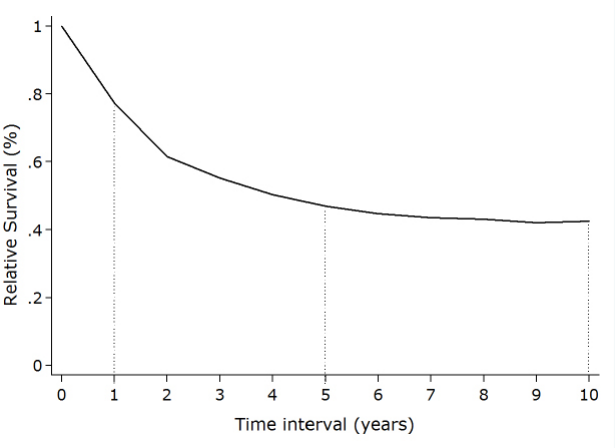
Relative Survival from invasive cervical cancer in Cali- Colombia, 1992-2001

## Survival and trends in clinical stage

Table 3 shows the relative survival from cervical cancer by histological type and clinical stage. About 63% of the squamous cell carcinomas and about 45% of the adenocarcinomas were diagnosed in stages II to IV. It should be noted that for 30% of the squamous cell carcinomas and for 40% of the adenocarcinomas no information on clinical stage was available.

**Table 3:**
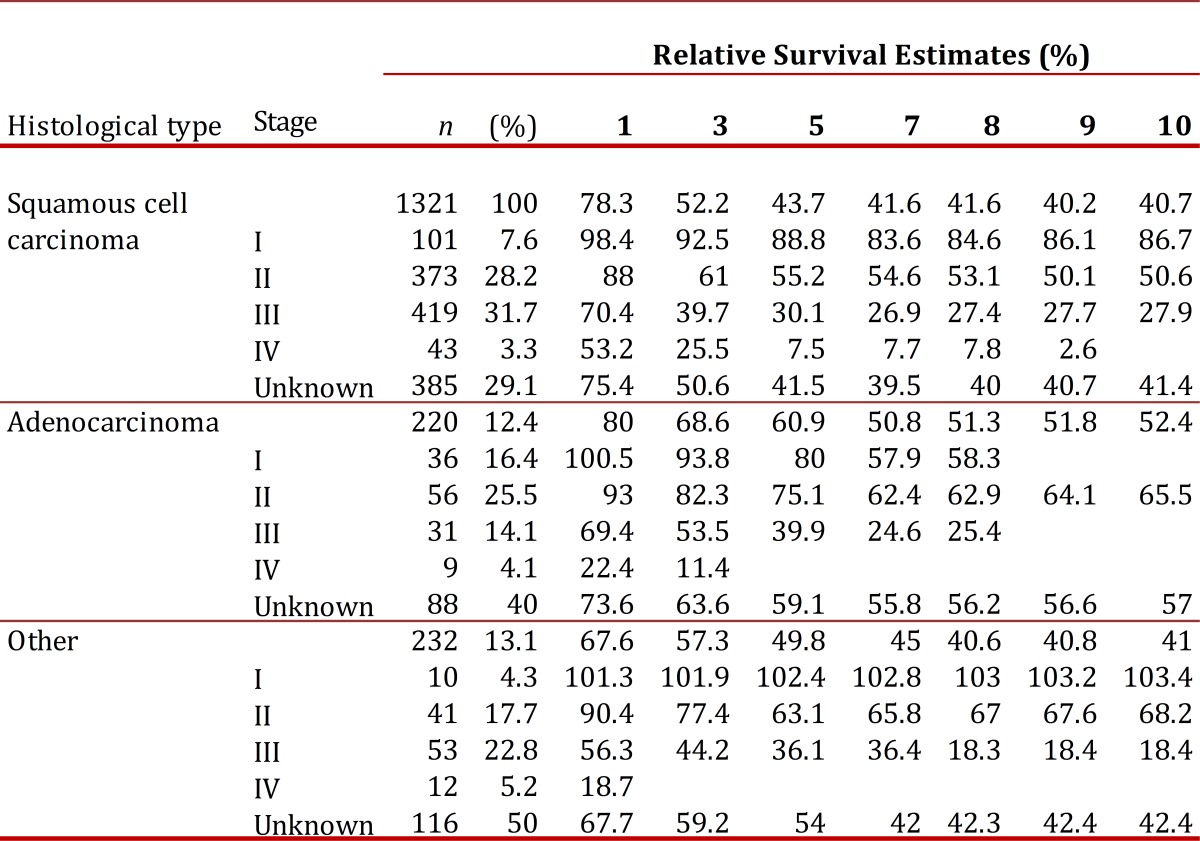
Cali, Colombia. Cervix Uteri Cancer Invasive: Number of cases and Relative Survival estimates (%) by Stage and Histological type.

The 5-year survival for stage I was 89% for squamous cell carcinoma and 80% for adenocarcinoma; conversely, for stage III it was 30% for squamous cell carcinoma and 40% for adenocarcinoma. Similar behavior was observed for the group labeled other histological types.

Figure 2 shows the Kaplan-Meier survival estimates for invasive cervical cancer during a 10 year period. Survival decrease rapidly during the first 4 years with half of the women dying by 3.4 years, but from year 6 they tend to stabilize.

## Etiology

One of us has had the privilege of being one of the scientists that participated in the discovery of HPV as the main cause of cervical cancer and in the application of this knowledge to the prevention of this cancer ^8^. HPV natural history studies have now revealed that HPVs are the commonest of the sexually transmitted infections in most populations. 

Most HPV exposures result in spontaneous clearance without clinical manifestations and only a small fraction of the infected persons, known as chronic or persistent carriers, will retain the virus and progress to precancer and cancer. Formal epidemiological evidence of an association between HPV and cervical cancer was lacking until the early 1990s ^9^. Molecular characterization and cloning of the first HPV types in the 1980s made possible the development of hybridization assays to look for HPV gene fragments in human tissue.

Using the first HPV hybridization assays developed and later on the PCR-based hybridization assays the Dr N Munoz & colleagues at International Agency for Research on Cancer (IARC) undertook the following fundamental molecular epidemiological studies to investigate the role of HPV in cervical cancer:

### 1- Case-control studies

The pioneering study of this program was carried out in Spain and Colombia ^10^ ^,^ ^11^ ^. ^In these two countries with contrasting cervical cancer rates, Spain with one of the lowest incidence and Cali with a high incidence, the first population-based case-control studies on HPV and cervical cancer were carried out; Exposure to HPV was measured using the three hybridization assays developed at that time). The population-based Cancer Registry of Cali was fundamental in the identification of the incident cases of cervical cancer diagnosed during the study period in this city. The results of these studies have been considered as the first unequivocal molecular epidemiological evidence of the causal association between HPV and cervical cancer ^10^. Similar studies were subsequently implemented in 9 other countries (Algeria, Brazil, India, Mali, Morocco, Paraguay, Peru, Thailand and the Philippines). In these 12 countries around the world we studied a total of 2,500 women with cervical cancer and 2,500 control women without cancer. These women were interviewed using a standardized questionnaire to elicit information on risk factors for cervical cancer and underwent a gynecological examination to collect cervical cells from the tumours and normal cervices for the detection of HPV DNA of 30 HPV types that infect the genital tract. The prevalence of HPV DNA was over 95% in the tumors cells of women with cervical cancer and it ranged from 5 to 20% in normal cervical cells of control women. These prevalences correspond to Odds Ratios (ORs) of over 100 indicating a very strong association between HPV and cervical cancer. The magnitude of the ORs allowed an epidemiological classification of 15 HPV types as carcinogenic or high-risk types, 12 as low-risk types and 3 types as probably carcinogenic ^12^. This classification has been reviewed in 2009 by the IARC leaving the following 12 HPV types as class 1 or carcinogenic: HPV 16, 18, 31, 33, 35, 39, 45, 51, 52, 56, 58 and 59 HPV68 as class 2A or probably carcinogenic and 12 other types as class 2B or probably carcinogenic.^13^

Our case-control studies also allowed the identification of the following cofactors that acting together with HPV increase the risk of progression from HPV persistent infection to cervical cancer: tobacco, high parity, long term use of oral contraceptives and past infections with herpes simplex type 2 and Chlamydia trachomatis.^14^ In addition, they contributed to establish the important role of male sexual behavior in the risk of developing cervical cancer.^15^

### 2- Survey of HPV types in invasive cervical cancers

Over 1,000 women with invasive cervical cancer from 22 countries around the world including Colombia were included in this study. HPV DNA detection with PCR-based assays revealed that 99.7% of the cases were HPV-positive. This finding led us to propose for the first time that HPV was not only the main cause of cervical cancer, but also a necessary cause.^16^ No other cancer has been shown to be a necessary cause.

The above two studies made possible to estimate the proportion of cervical cancer cases attributable to the main HPV types in the various geographical regions. They showed that HPV 16 and 18 account for 70% of cervical cancer and the 8 most common types (HPV 16, 18, 45, 33, 31, 52, 58 and 35) account for about 90% of cervical cancer.^17^These estimates have been confirmed in a larger survey including over 10,000cases of invasive cervical cancer from 43 countries around the world. (de Sanjose et al) and are being used to estimate the impact of preventive strategies based on HPV.^18^

### 3- Implications for Prevention

The demonstration that infection with certain types of human papillomavirus (HPV) is not only the main cause but also a necessary cause of cervical cancer has led to great advances in the prevention of this disease on two fronts:(i) Primary prevention by the use of prophylactic HPV vaccines; and (ii) secondary prevention by increasing the accuracy of cervical cancer screening.

(i) In primary prevention by the use of prophylactic HPV vaccines; Two safe and efficacious prophylactic HPV vaccines have been developed using viral like particles (VLPs); the quadrivalent vaccine (Gardasil) contains VLPs of HPV 16 and 18, responsible for about 70% of cervical cancers, a considerable proportion of other genital cancers and cancers of the oral cavity and pharynx and VLPs of HPV6 and 11 that cause about 90% of genital warts and recurrent respiratory papillomatosis (RRP). The bivalent vaccine contains only VLPs of HPV16 and 18. In young women (15- 26 years old) who have not been exposed to HPV, both vaccines have been shown to prevent high-grade precancerous lesions of the cervix (CIN2/3) with efficacies close to 100%, and this protection has been shown to last at least 7-8 years. ^19^ ^-^ ^20^ The quadrivalent vaccine has been shown, to have in addition, a high efficacy for the prevention of high-grade precancerous lesions of the vulva, vagina, and genital warts and of the anus in men.^19^ ^,^ ^21^ The bivalent vaccine has been reported to have a high efficacy for the prevention of persistent anal HPV infection in women.^22^ Some degree of cross-protection for HPV types phylogenetically related to HPV 16 and 18 have been reported for both vaccines. Pre- and post-licensure studies have shown that both vaccines are safe and well tolerated.

The World Health Organization (WHO) recommends a 3-dose vaccine schedule, completed over the course of 6 months, for a likely primary target population of girls within the age range of 9 or 10 years through 13 years.^23^

The main limitation of both vaccines is that they protect against cancers produced only by HPV 16 and 18 (about 70% of cervical cancers), and since they are prophylactic, they do not have any effect on established HPV infections or their associated lesions, (they do not have therapeutic effect). Therefore, they do not preclude the need of screening.

Both vaccines have been licensed in about 120 countries. By 2011, national HPV vaccination programs had been introduced in over 35 countries, in the developed world. The United States, Australia, and Canada were among the first countries to introduce HPV vaccine into their national immunization programs in 2006-2007 , and coverage is higher in Australia and Canada (over 80%), where the administration of the vaccine is school-based. The main challenges for the introduction of the HPV vaccine in immunization programs in low and middle income countries are: their high price and the lack of infrastructure to reach adolescents and immunize them with 3 doses. Great advances have been made recently in both fronts; the GAVI Alliance (GAVI) announced in the fall of 2011 that it will provide HPV vaccines for the poorest countries (GAVI- eligible countries in Latin America: (Haiti and Nicaragua); the company producing the quadrivalent vaccine has offered to GAVI a price of $5 dollars per dose.^24^

For middle income countries, manufactures are offering lower prices based on negotiations such as those conducted by the PAHO Revolving Fund. Through this fund, Latin American countries may acquire the vaccine at around $14 US dollars per dose as opposed to the initial commercial price of about $120 US dollars per dose. Concerning vaccine delivery, pilot projects have shown that highest coverage is reached through school-based programs, and a sub-analysis within the Guanacaste HPV vaccine trial in Costa Rica has revealed that less than 3 doses may confer good protection ^25^; schedules with less than 3 doses will facilitate high coverage. Preferably, HPV vaccines should be introduced as part of a coordinated strategy to prevent cervical cancer and should not undermine effective cervical cancer screening programs in those countries where these programs are in place. In most developing countries where effective screening programs do not exist or will be very difficult to implement, the ideal strategy will be based on vaccination of adolescent girls.

In Latin America only 5 countries have introduced the vaccine in their national immunization programs: Panama, Mexico, Peru, Argentina and Colombia. In Colombia, the HPV vaccine is being offered to girls in 4th year of primary school (9-10 years old).(ii) In secondary prevention by increasing the accuracy of cervical cancer screening. Well organized screening programs have been successful in reducing cervical cancer incidence and mortality in developed nations, but they have been unsuccessful in the great majority of developing countries.^26^The main reasons for the lack of impact of cytology-based screening programs in Colombia have been identified. They include poor cytology quality and lack of follow-up and treatment of 30-40% of women diagnosed with high-grade cervical lesions.

Several clinical trials have shown that HPV DNA detection assays are more sensitive but a bit less specific than cytology for detection of high grade precursor lesions of the cervix (CIN2/3) and suggest that they should be used as primary screening test instead of cytology. ^27^ A cluster randomized trial in India has reported that a single round of screening with HPV test was followed by a 50% reduction in mortality from cervical cancer in women 30 to 59 years old after 8 years of follow-up, as opposed to not effect of cervical cytology or screening with VIA.^28^ The lower specificity of HPV-based screening as compared with cytology-based screening leads to the possibility of over treatment of cervical lesions, that if left untreated, will regress. Research efforts are centered now in finding the best way to triage women found positive for HPV; various biomarkers including type specific HPV 16/18, RNA, p16 are being evaluated. Cost-effectiveness evaluation of conventional cervical cytology and HPV testing for cervical screening in Colombia have shown that HPV testing every 5 years in women over 30 years of age is a cost-effective strategy, provided that the cost of the HPV test is less than 31 US dollars.^29^ In addition, a demonstration project in very low income populations near Bogota has shown that screening using visual inspection with acetic acid (VIA) and Lugol's iodine (VILI) is more sentive but less specific than cytology or VIA alone and provides bases to implement see and treat strategies in very deprived populations.^30^ The above results led to the ministry of health of Colombia to approve screening strategies based on scientific evidence and to include the use of the HPV test as primary screening test in the social security system, and to expand the VIA-VILI screening program to 5 other very low-resources areas in Colombia. (Amazonas, Buenaventura, Caquetá, Guajira, Tumaco). Similar decisions have been taken in Mexico that decided to formulate a comprehensive strategy for the control of cervical cancer including HPV-based screening and HPV vaccination of all 11 years old girls.^31^

It is hope that a fast and inexpensive HPV test (CareHPV at less than $5 dollars) developed with funds from the Gates foundation will be shortly commercially available^32^

In conclusion, the main hope to reduce the burden of cervical cancer in Colombia and Latin American countries lies in the introduction of the prophylactic HPV vaccine to adolescent girls and in the introduction of the HPV assay as primary screening test. ^33^
